# Microalgae Cultivated in Industrial Wastewater as Agricultural Bioinputs: Technical and Life Cycle Assessment to Support Sustainable Production

**DOI:** 10.1021/acsomega.5c08477

**Published:** 2025-11-24

**Authors:** Karoline Matiello Almeida, Bianca Barros Marangon, Vinícius José Ribeiro, Jackeline de Siqueira Castro, Juscimar da Silva, Edson Marcio Mattiello, Andreia Aparecida de Sousa Silva, Eduarda Cristina Moreira Silva, Maria Lúcia Calijuri

**Affiliations:** † Department of Soils and Plant Nutrition, 28120 Federal University of Viçosa (Universidade Federal de Viçosa), Viçosa, Minas Gerais 36570-900, Brazil; ‡ Department of Civil Engineering, Center for Advanced Research in Microalgae, 28120 Federal University of Viçosa (Universidade Federal de Viçosa), Viçosa, Minas Gerais 36570-900, Brazil; § 67838 Brazilian Agricultural Research Corporation − EMBRAPA Vegetables, Brasília, Distrito Federal 70359-970, Brazil

## Abstract

This study evaluated the technical and environmental feasibility of using wastewater-cultivated microalgae biomass as a nutrient source for tomato plants. A field experiment tested different foliar application doses of microalgae biomass (control, 0.5%, 2.5%, 5%, and 10%) on tomato plants. Postharvest analysis of eight plant and fruit parameters showed no significant differences among treatments. Environmental feasibility was assessed through life cycle assessment, comparing a baseline scenario (mineral fertilizer) with an alternative scenario in which microalgae biomass was used as both a nutrient and water source. The Ecoinvent database and ReCiPe 2016 methodology were applied at both midpoint and end point levels. The alternative scenario demonstrated reduced environmental impacts across all 18 midpoint categories, including substantial reductions in water consumption (107.43%) and ionizing radiation (53.54%). At the end point level, the baseline scenario had 23.05% higher impact in the human health category than the alternative.

## Graphical abstract

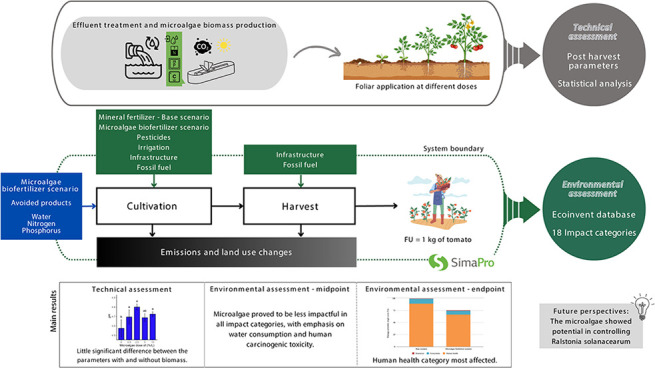

## 1 Introduction

Due to the increase in population and the consequent demand for food, synthetic chemical fertilizers have been extensively used in traditional agriculture.^1^ Nutrient deficiency in the soil is one of the critical factors limiting crop growth and further driving the use of these products.^2^ This conventional agricultural model provides nutrients to crops but contributes to the degradation of soil quality and the disruption of the microbial balance.^3^ Furthermore, the production of synthetic fertilizers leads to high electricity consumption, resulting in intense carbon emissions into the atmosphere.^4^ Therefore, the adoption of good management practices aimed at more environmentally balanced models is crucial for the development of modern agriculture.

In this context, microalgae-based bioinputs stand out as an alternative source of nutrients, phytohormones, and biocontrol agents to ensure food supply for a growing global population and achieve the United Nations Sustainable Development Goals.^5–7^ Microalgae can also be cultivated during wastewater treatment, contributing to its purification and, consequently, to the protection of the environment.

Biofertilizers are products containing living microorganisms or biologically active substances that promote plant growth by increasing the availability of nutrients or stimulating physiological processes, for example, *Rhizobium*, *Azospirillum*, and microalgae-based inoculants. Foliar fertilizers, in turn, are nutrient formulations applied directly to the leaves to correct deficiencies or stimulate plant metabolism through rapid nutrient absorption.^8^

With the exception of biofertilizers, other mechanisms of action of microalgae as agricultural bioinputs have not been extensively investigated. These include foliar fertilizers and bactericidal, pesticidal, and fungicidal effects,^9–12^ highlighting an increasing need for advancements in the technical field. Moreover, the environmental impact of these emerging biotechnologies needs to be explored in order to understand their life cycle and the contribution of each production stage, so that adjustments can be made as necessary.

Several initiatives have been developed to explore the use of microalgae as agricultural bioinputs.^4,9,11,12^ Recent projects in Brazil, supported by national research agencies, have investigated the production of biofertilizers and biostimulants from wastewater-grown microalgae, aiming to integrate nutrient recovery with sustainable agriculture. These studies reinforce the potential of microalgae to replace part of synthetic fertilizers while promoting circular bioeconomy strategies.^4^ However, despite these advances, few works have combined technical, environmental, and life cycle assessments (LCA) to evaluate the large-scale feasibility of using wastewater-grown microalgae as foliar or soil biofertilizers, highlighting an important research gap.

The manuscript in question results from the initial technical and environmental investigations conducted in the Cerrado, within the scope of the aforementioned project. Specifically, the soils in the Cerrado are naturally acidic and have low nutrient availability, requiring the application of fertilizer sources, whether foliar or directly to the soil, to make them productive. This increases production costs and may impact long-term sustainability.

Maureira et al.^13^ highlighted the environmental feasibility of open-field tomato production compared to greenhouse cultivation. Furthermore, Solimene et al.^14^ emphasized the need to adopt innovative approaches for sustainability, such as the implementation of closed-loop resource recovery systems and other innovative strategies to further enhance the sustainability of tomato production systems.

The production of chemical fertilizers is associated with the generation of pollutants that cause environmental impacts. Studies that carried out LCAs of similar systems identified the use of nitrogen fertilizers as a hotspot for pollutant emissions.^15,16^ When it comes to phosphorus, deposits must be mined and processed to transform the ore into bioavailable P fertilizer. The resulting waste may contain potentially toxic trace metals, and the process contributes to greenhouse gas emissions and eutrophication.^17^ Therefore, it is essential to seek alternatives for the more sustainable production and use of these essential inputs for modern agriculture.

Therefore, considering the potential of wastewater treatment plants as resource recovery units through microalgae cultivation and the application of this biomass as an agricultural bioinput, the present study aimed to (i) evaluate the application of microalgae as a foliar fertilizer for industrial tomato crops in the Brazilian Cerrado region at different doses; and (ii) provide insights, through the use of LCA, on the environmental impacts generated and avoided as a result of using the bioinput under investigation.

## 2 Material and Methods

### 2.1 Algal Biomass Production

The algal biomass was generated at the experimental area for wastewater treatment and biomass production at the Sanitation and Environmental Engineering Laboratory of the Federal University of Viçosa (UFV) in Minas Gerais, Brazil (UTM coordinates 722924 E, 7702003 S, zone 23 K). Located at an average altitude of 648 m, the municipality of Viçosa experiences an annual average rainfall of around 1221 mm and a mean yearly temperature between 19 and 20 °C, with an average relative humidity of 81%. According to the Köppen classification, the region’s climate is categorized as Cwa, a tropical altitude climate with hot, rainy summers and cool, dry winters.^18^

The microalgae biomass was cultivated as a byproduct of wastewater treatment from a meat processing facility, using high-rate algal ponds (HRAPs). This facility primarily produces sausages (such as salami and hams) and shredded desalted codfish. Industrial wastewater arises at different stages of the production process, notably from the disposal of cooking and cooling water from sausage production, desalting of cod, and from the cleaning of floors and equipment at the end of production cycles.

To ensure a biomass appropriate for the intended application, the wastewater underwent initial characterization. In this study, a nitrogen-rich effluent from a primary flotation unit was selected, with characteristics detailed in previous research.^4,19^

The pilot-scale HRAPs used for biomass production had the following specifications: width = 1.28 m, length = 2.86 m, total depth = 0.50 m, working depth = 0.30 m, surface area = 3.30 m^2^, and working volume = 1.00 m^3^ (Figure 1). The HRAPs were constructed with fiberglass, and the paddle wheels, made of stainless steel with six blades, were driven by a 1 hp electric motor. The rotation was reduced via a reducer connected to the motor and managed by an inverter (WEG CFW-08 series), maintaining a liquid velocity of 0.10 to 0.15 m s^–1^. These operational parameters align with similar studies utilizing HRAPs^20,21^ and provided effective mixing.

**Figure 1:**
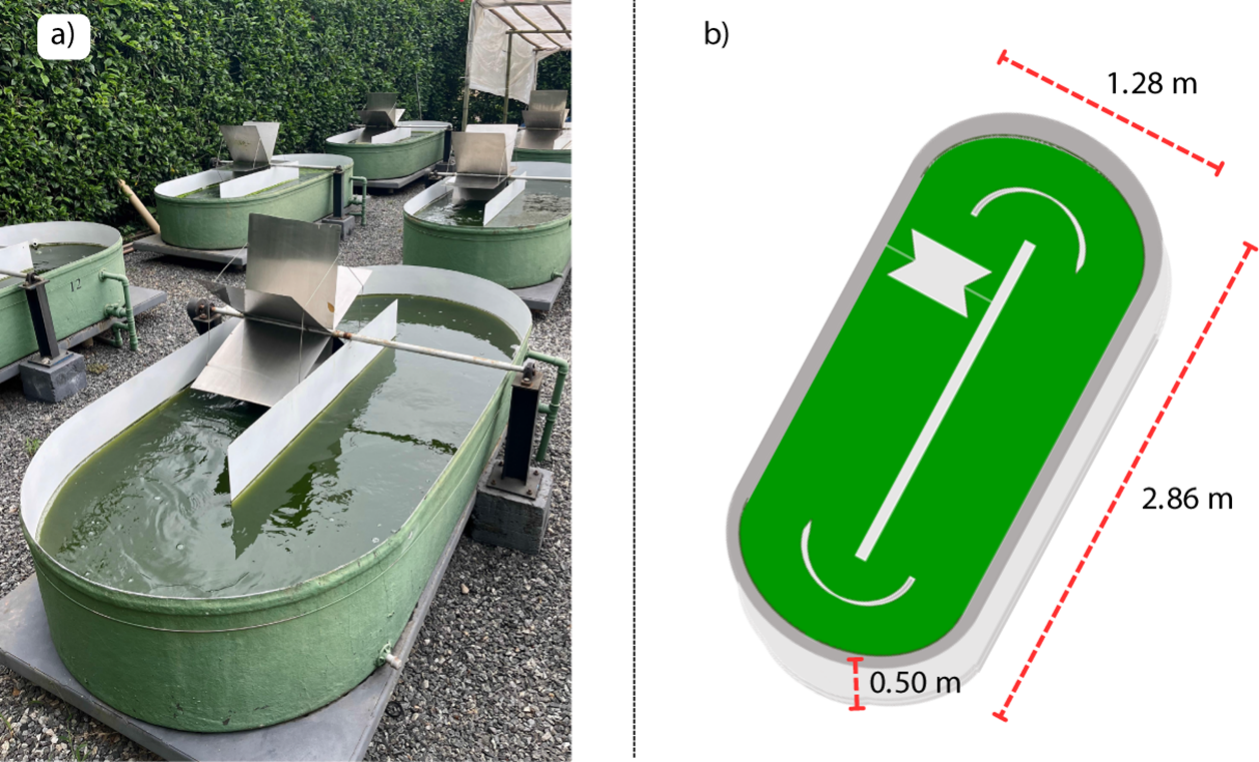
HRAP used for biomass production: (a) photograph of the pilot-scale high-rate algal ponds (HRAP) located in the experimental area (photograph taken by the authors); (b) schematic top-view drawing of the HRAP with dimensions.

The HRAPs were operated until the algal growth decay phase was reached, monitored through chlorophyll-*a* levels. After this period, paddlewheel rotation was stopped, and the biomass was collected via gravitational sedimentation. The concentrated material at the bottom of the HRAP was manually collected using plastic containers after wastewater disposal. Then the biomass was concentrated by a high-speed refrigerated centrifuge (Thermo Scientific Multifuge X3R, rotor F14-6 × 250 LE, 6 × 250 mL), at 10,000 rpm (∼15,300*g*) for 3 min. Biomass samples were then lyophilized for characterization analyses.

### 2.2 Algal Biomass Characterization

The microalgae biomass used for foliar application was characterized in terms of phytoplankton community, based on Komarek and Fott^22^ and Parra et al.,^23^ based on the methodology described by APHA,^24^ Utermöhl,^25^ and Wetzel and Likens.^26^

The microalgae biomass biochemical composition and the macro- and micronutrient was also identified. The lipid content was determined gravimetrically after cell disruption using a cell-crushing mill (Tecnal TE-099) and extraction using the Soxhlet method^27^ using the Tecnal TE-044-/50 fat extractor. Protein content was indirectly determined using the Total Kjeldahl Nitrogen (TKN) method, according to the Standard Methods for the Examination of Water and Wastewater^24^ with a conversion factor of 6.25. Carbohydrates in the biomass were determined by difference. Ash content was conducted according to ASTM D3172 (ASTM, 2021). The carbon, hydrogen, and nitrogen (CHN) content of the microalgae biomass was determined using a Vario Micro Cube Elemental Analyzer. Helium and oxygen were used as the carrier and ignition gases, respectively. Macro- and micronutrient contents were analyzed using Inductively Coupled Plasma Optical Emission Spectrometry (ICP-OES).

### 2.3 Experimental Conditions

The trial was conducted at the experimental field of Brazilian Agricultural Research Corporation (EMBRAPA) Vegetables (Brasília, DF, Brazil), located at 15°56′ S and 48°08′ W, at an altitude of 997.6 m. According to the Köppen climate classification, the region’s climate is characterized as tropical savanna (Aw), with average maximum and minimum temperatures of 28.3 and 12.9 °C, respectively. The annual evaporation in a Class A Pan is approximately 2000 mm, with a daily average of 5.6 mm. Relative air humidity ranges from 70% during the rainy months to 10% in the dry months. The rainy season occurs from October to April, with an average annual precipitation of 1400 mm.

The experiment was conducted between June and October 2023, using industrial tomato crop, cultivar HEINZ 7885. A drip irrigation system was adopted, with emitters spaced 20 cm apart. Other cultural practices were performed mechanically, and the harvest was carried out manually. The soil in the experimental area, classified as clayey, was prepared through liming and fertilization according to the recommendations from soil analysis.

The experimental design followed a randomized block layout with five treatments, including four doses of freeze-dried and concentrated algae and a control. All treatments received the same basal mineral fertilization, which was applied according to soil analysis recommendations. The freeze-dried and concentrated algae were used as a foliar biofertilizer supplement, applied at different concentrations (0.5%, 2.5%, 5%, and 10%) in addition to the mineral fertilization.

The treatments were defined as follows: T1—Control (foliar application of water); T2—Algae 0.5%/L; T3—Algae 2.5%/L; T4—Algae 5%/L; and T5—Algae 10%/L. Drip irrigation was used to supply water and mineral nutrients to all treatments, ensuring uniform soil moisture and nutrient availability throughout the experiment. In addition to this irrigation, foliar applications of freeze-dried and concentrated microalgae were performed at concentrations of 0.5%, 2.5%, 5%, and 10%. Each foliar spraying corresponded to approximately 2 L of solution per plot, applied in the morning at three crop stages: seedling establishment, full flowering, and fruit maturation. Therefore, drip irrigation ensured consistent water supply to all plots, while foliar spraying represented an additional treatment aimed at delivering the microalgae biofertilizer directly to the leaf tissues. The experiment was conducted using a double-row system, with a spacing of 0.5 m between rows, in plots measuring 5 m in length each. Each row had a total length of 50 m, divided into 10 plots per row, with a spacing of 1.5 m between double rows (Figure 2).

**Figure 2:**
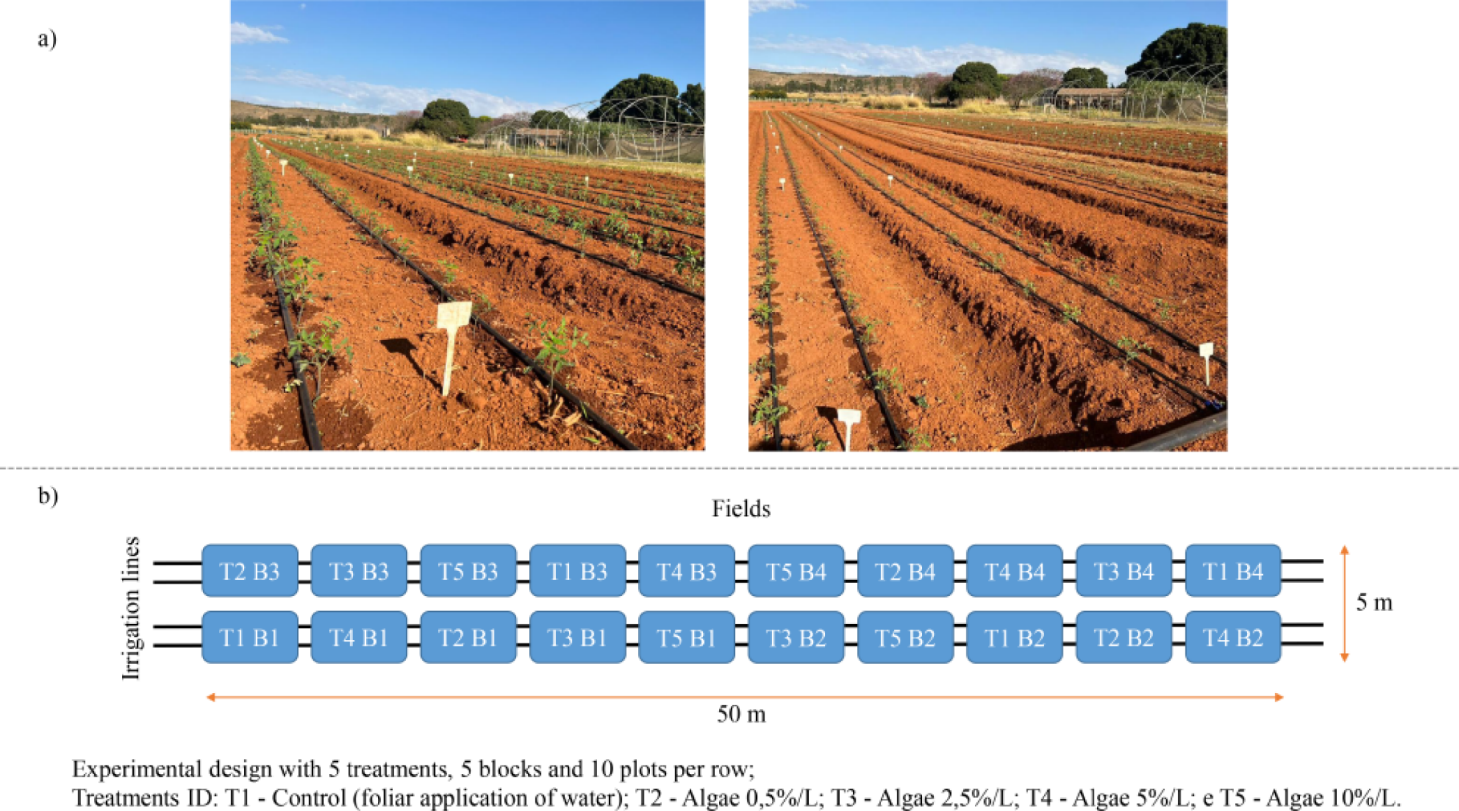
Experimental field: (a) photograph of the site where foliar application of microalgae extract was carried out (photograph taken by the authors); (b) schematic layout of block design with dose distribution.

During the trial, an infestation of bacterial wilt caused by *Ralstonia solanacearum* was detected at the flowering stage of the crop. The disease led to flower abortion and the anticipation of production, resulting in the loss of several plants within the plots. This factor may have contributed to the lack of significant differences between treatments.

### 2.4 Plant Analysis

The chlorophyll content was measured using a SPAD-502 Plus chlorophyll meter (Konica Minolta, Osaka, Japan). Measurements were taken on the leaf blade of four plants per plot, providing immediate results for the estimated chlorophyll content. The harvest was carried out manually 120 days after planting, collecting four plants per plot. The tomatoes were counted, weighed, and subsequently measured for diameter (mm) and length (mm). Postharvest parameters were also analyzed, including Brix, pH, and total titratable acidity, following the methodology described by Moretti.^28^

### 2.5 Statistical Analysis

All data were subjected to analysis of variance (ANOVA). The Shapiro–Wilk and Levene tests were applied to assess residual normality and homogeneity of variances. Treatments were compared using the Tukey test (*p* < 0.1). All statistical tests were performed in R version 4.2.2 (R Core Team , 2022).

### 2.6 Life Cycle Assessment (LCA)

The LCA modeling was conducted using SimaPro software v.9.6.0.1 PhD. The International Organization for Standardization (ISO) standards ISO 14040 and 14044 were followed. The LCA was carried out in four stages: (i) goal and scope definition; (ii) life cycle inventory (LCI); (iii) life cycle impact assessment (LCIA); and (iv) results interpretation.^29,30^

The goal of the LCA was to compare the potential environmental impacts of conventional/commercial tomato production with nutrient supply from microalgae biomass. This was done to estimate the potential for reducing environmental impacts provided by incorporating microalgae as a partial nutrient source for the crop by mixing the biomass with conventional fertilizers, since microalgae alone cannot meet all the crop’s needs. For this purpose, the data set *“Tomato, processing grade (IT)|tomato production, processing grade, open field|APOS.S”* from the Ecoinvent v3.8 database was used. This process represents the production of 1 kg of processing-grade tomatoes in Emilia Romagna, Italy. Processing-grade tomatoes are used for industrial processing into pulp, juice, or paste.

The system boundary, from cradle to gate, starts with soil cultivation after the harvest of the previous crop and ends with harvest and transport to the farm gate. Storage is not included. This is a conventional/commercial tomato crop. Therefore, activities such as machinery operations (soil cultivation, fertilization, pesticide application, harvesting, and on-farm transportation), corresponding infrastructure, fuel use, and storage buildings are included. Additionally, direct field emissions and land-use changes are considered. Irrigation, seedling inputs, fertilizers, and pesticides, as well as packaging for fertilizers and pesticides, are also accounted for. The absorption of heavy metals by the crop is considered as well.

It is important to note that the tomato crop data set available in the Ecoinvent database includes a broader range of inputs and activities than those considered in the experiment conducted in this study. While the experiment focused on assessing the effect of foliar application of microalgae, the LCA aimed to estimate the potential reduction in environmental impacts achieved by combining microalgae with mineral fertilizer. This approach highlights the benefits of reducing the reliance on mineral fertilizers through the incorporation of an organic nutrient source.

The reference flow was the average yield of 71 t ha^–1^ for the year 2011, obtained under irrigated conditions (total water quantity of 1800 m^3^ ha^–1^). The input of mineral nitrogen–phosphorus–nitrogen, phosphorus, and potassium (NPK) fertilizer was 130–130–200 kg ha^–1^. The total active ingredients (a.i.) applied as pesticides amounted to 21.8 kg a.i. ha^–1^. In the baseline scenario (commercial production), no organic fertilizer was applied. In the modified scenario, microalgae biofertilizer containing NPK in the proportion of 5.72–1.44–0.46 (Table 1) was incorporated to supply part of the tomato’s nutritional demand. For this purpose, it was assumed that the N, P, and K contained in the microalgae biomass (Table 2) would be fully available to the plants, while the remaining nutrient demand would continue to be supplied by mineral fertilizers. Additionally, the application of wet biomass was considered, providing both water and nutrients to the crop. For this, irrigation with treated wastewater containing 770 mg of biomass per liter was considered.^31^ In this way, the crops’ water requirements could be met with treated wastewater, thereby reducing the consumption of freshwater resources.

**Table 1:** LCI to Produce 1 kg of Tomato (Tomato, Processing Grade)

Input	Ecoinvent process	Applied to the tomato (Ecoinvent)	Avoided by the microalgae		Input to produce microalgae
N	Inorganic nitrogen fertilizer, as N {RoW} nutrient supply from urea|APOS, S	0.001831 kg N	0.001125 kg N	61.43%	-
P	Inorganic phosphorus fertilizer, as P205 (RoW] nutrient supply from monoammonium phosphate|APOS, S	0.001831 kg P	0.000280 kg P	15.30%	-
K	Inorganic potassium fertilizer, as K20 (RoW} nutrient supply from potassium chloride|APOS, S	0.002817 kg K	0.000090 kg K	3.21%	-
Water	Tap water {RoW}|tap water production, underground water without treatment|APOS, S	0.025352 m^3^ water	0.025352 m^3^ water	100.00%	-
Electricity	Electricity, low voltage (RoW| electricity voltage transformation from medium to low voltage|APOS, S	-	-	-	0.002147 kW h

**Table 2:** Characterization of Algal Biomass

Biochemical composition (wt %)	
Lipids (total)	16.11
Proteins	36.50
Carbohydrates^a^	28.84
Ash	18.55
Macro and micronutrient content (wt %)	
C	40.66
N	5.72
P	1.44
Ca	1.36
Na	0.70
Mg	0.68
S	0.56
K	0.46
Fe	0.28
Zn	0.02
Mn	0.01

a Obtained by difference (carbohydrates = 100% – (lipids + proteins + ash)).

The treatment of wastewater is the responsibility of the generator; therefore, for the separation of microalgae and treated wastewater, only the electricity demand for harvesting this biomass was considered, amounting to 0.11 kW h kg^–1^ of algae for the dissolved air flotation unit.^32^

Table 1 presents the changes made to the life cycle inventory (LCI) of the process *Tomato, processing grade (IT)|tomato production, processing grade, open field|APOS.S* from the Ecoinvent 3.8 database to obtain the scenario with the application of microalgae biomass as fertilizer. For the baseline scenario, the original process from Ecoinvent 3.8 was maintained. In the scenario that considered microalgae as a nutrient source and treated wastewater for irrigation, these inputs were modeled as avoided products. All other inputs, outputs, and emissions from the ecoinvent database process remained unchanged between scenarios and, for this reason, are not reported. The reference flow has the functional unit of producing 1 kg of tomatoes.

The LCIA method used was ReCiPe 2016 v.1.1, hierarchized at the midpoint and end point levels. This method utilizes global impact mechanisms. The results were characterized and classified into the 18 Midpoint impact categories provided by ReCiPe. These categories include global warming, stratospheric ozone depletion, ionizing radiation, ozone formation (human health and ecosystems), fine particulate matter formation, terrestrial acidification, freshwater and marine eutrophication, terrestrial, freshwater, and marine ecotoxicity, human carcinogenic and noncarcinogenic toxicity, land use, mineral resource depletion, fossil resource depletion, and water consumption.

At the end point level, potential environmental impacts were obtained in terms of human health (expressed in disability-adjusted life years and the number of years lived with disabilities, DALY), ecosystems (expressed in species loss per year in a specific area, species.yr), and resources (expressed in surplus costs of future resource production over infinite time due to resource scarcity, USD2013).

Subsequently, these results were normalized into ecopoints, considering the reference factor of the method (the average global pressure applied to the environment by an individual in 2010).^33^ Based on these results, it was possible to identify the main differences, in terms of potential environmental impacts, arising from the application of microalgae biofertilizer in tomato production and provide insights to improve the environmental performance of this crop’s production.

## 3 Results and Discussion

### 3.1 Algal Biomass Characterization

The microalgae biomass was primarily composed of Tetradesmus obliquus (72%) and *Chlorella vulgaris* (23%) in terms of relative abundance.

The biochemical composition and the macro- and micronutrient content of the biomass are presented in Table 2.

The biochemical and elemental composition of the biomass obtained was similar to that of Castro et al.^34^ and Pereira et al.,^35^ in meat processing wastewater.

### 3.2 Technical Evaluation of Tomato Production under the Effect of Microalgae Biomass

The postharvest parameters of plants and fruits were analyzed and discussed to evaluate the effects of different treatments. These parameters provide valuable insights into the quality, nutritional content, and shelf life of the harvested tomatoes, as well as the overall health and productivity of the plants. By examining these factors, it is possible to assess the effectiveness of microalgae biomass applications in enhancing crop performance and determine any potential benefits for sustainable agricultural practices. Statistical analyses were applied to identify significant differences between treatments, highlighting key outcomes and areas for future research.

The pH did not show statistically significant differences between the treatments with microalgae application. The control treatment (T1) showed statistically similar results to the T4 treatment. The samples that received foliar application of microalgae showed higher pH values compared to those without application (T1). This increase in pH ranged from 2.35% (T4) to 5% (T3) in relation to the control, suggesting a possible reduction in the acidity of the samples treated with microalgae (Figure 3). Such an increase is a desirable characteristic, since higher pH values are often associated with greater sensory acceptance of the product.^36^ The values obtained are in accordance with those reported in the literature, which vary between 4.87 and 5.44 ^37^ and between 4.37 and 4.58.^38^

**Figure 3:**
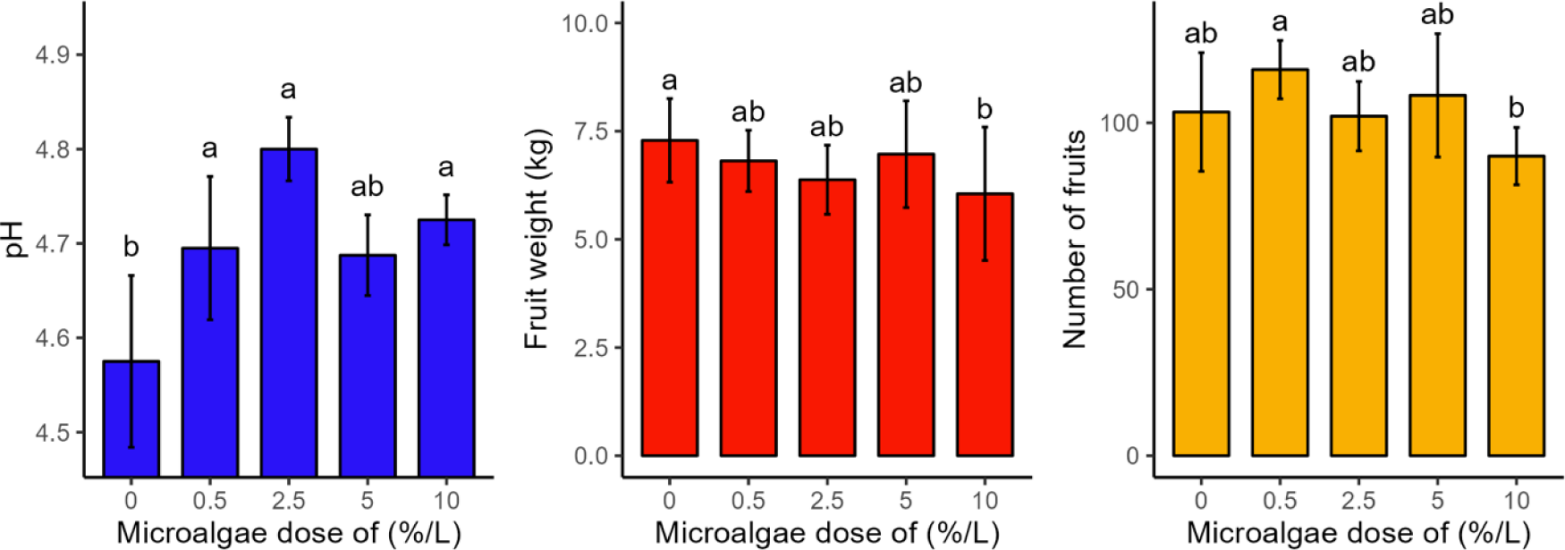
(a) pH of fresh tomatoes; (b) fruit weight and (c) number of fruits (*n* = 4) under different concentrations of microalgae. Equal letters indicate that there was no statistical difference between the means at the 10% level of the Tukey test. Bars indicate standard deviation.

The weight (kg) did not differ statistically between the treatments with microalgae application. Furthermore, the control (T1) presented statistically similar results to the treatments with microalgae application, except for the treatment with a higher dose of the product (T5). The use of microalgae-based biofertilizers has been explored as a sustainable alternative in several crops. García-Orellana et al.^39^ investigated the effect of *Chlorella sp*. and *Scenedesmus sp*. biofertilizer on basil (*Ocimum basilicum* *L.*) cultivation and observed that the application of this input did not have a significant effect on plant height compared to the control treatment, without biofertilizer. In another study, Coppens et al.^40^ compared the performance of organic, microalgae (*Nannochloropsis oculata*) and conventional fertilizers in tomato cultivation. The authors found that the use of microalgae fertilizers resulted in lower fruit yield compared to inorganic and organic systems, however, an increase in fruit quality was observed, with an increase in sugar and carotenoid contents. Dagnaisser^41^ evaluated the application of microalgae biomass in the production of arugula (*Eruca vesicaria* *L.*), finding that the use of urea as a conventional nitrogen source resulted in higher production when compared to treatment with microalgae.

Regarding the number of fruits, the control treatment (T1) did not present a statistical difference in relation to the treatments with microalgae application, except in comparison to the treatment with the highest dose of the product (T5), which showed a 14% reduction in comparison to the control. Comparing the performance of organic, microalgae (*Nannochloropsis oculata*) and conventional fertilizers in tomato cultivation, Coppens et al.^40^ also found no significant difference in the number of fruits between treatments. Pooja et al.^42^ provided, as a biofertilizer for tomato (*Solanum lycopersicum*) cultivation, municipal wastewater treated in an outdoor open syntax tank with *Chlorella vulgaris* and found an increase in the growth rate and fruit yield. Plants grown with treated wastewater produced fruits in quantities and with weights almost equivalent to those obtained when chemical fertilizer (urea) was used. Furthermore, the nitrate levels in tomatoes grown with treated wastewater were lower than those in tomatoes grown with chemical fertilizer, indicating lower toxicity to human health, which is an advantage of the biofertilizer.

The average production observed in our study was 26 fruits per plant (Table S1). Similarly, Turnes,^43^ when evaluating different pruning methods in tomato cultivation of the Valerin cultivar, reported the occurrence of bacterial wilt caused by *Ralstonia solanacearum* during the experiment, where he obtained an average production of 27 fruits per plant. In contrast, Lima^44^ observed that tomato plants infested by *Ralstonia* had an estimated productivity of 46.5 t ha^–1^. In comparison, in our trial, the estimated productivity was 22.3 t ha^–1^ (Table S1). The expected productivity in industrial tomato crops without Ralstonia infestation is approximately 90 t ha^–1^.^45^

Infestation by *Ralstonia* compromises both the quantity and quality of the fruits. The disease is difficult to control, and once the pathogen infects the planting area, the bacteria spread very quickly. The economic impact can be devastating, since entire cultivation areas can be affected, leading to a significant reduction in production.^46^ In this sense, it is assumed that the disease had a major impact on the final production result of the experiment, and may be one of the factors responsible for the low productivity.

There was no significant difference in °Brix between the treatments, with values ranging from 3.9 to 4.20 °Brix. These values are consistent or even higher when compared to some values found in the literature, which range from 4.04 to 4.38 °Brix,^47^ 2.34 to 3.67 ^48^ or 4 to 4.2 °Brix.^49^ The soluble solids content (TSS) given in °Brix is one of the main factors that determine the yield of processed tomato pulp. The higher the TSS content, the higher the yield at the industrial level, where for each increase of 1 °Brix in the raw material, there is an approximate increase of 10 to 20% in the industrial yield.^50^ Genetic factors of the cultivar and crop management, such as fertilization, temperature and irrigation, can influence its value.^51^

Additional variables, such as SPAD index, fruit length and diameter, pulp, and citric acid, did not show statistical differences between treatments. Studying the seaweed extract *Ascophyllum nodosum* *(L.) Le Jolis*, Koyama et al.^52^ found that a dose of 0.3% for protected and field cultivation, applied every 15 days, increased production but did not alter fruit characteristics or plant vegetative growth. Similarly, the application of 2% microalgae biomass to the soil led to greater soil basal respiration, microbial biomass carbon, and β-glucosidase, acid phosphatase, and arylsulfatase enzymatic activity; however, it did not promote greater growth of corn (*Zea mays* *L*).^53^

Although treatment effects were not statistically significant, our working hypothesis was that foliar applications of wastewater-grown microalgae, containing nutrients and bioactive compounds, could biostimulate tomato plants and improve postharvest quality. The lack of significance may reflect: (i) limited foliar bioavailability of whole biomass, (ii) suboptimal dose/timing relative to phenological stages, (iii) masking by uniform mineral fertilization (ceiling effect), (iv) batch-to-batch variability in biomass composition, (v) environmental variability and limited statistical power, and (vi) the absence of uptake-enhancing coformulants. Future trials will test refined doses and schedules, evaluate standardized extracts and adjuvants, and increase experimental power; where disease mitigation is hypothesized, dedicated assays under controlled inoculation will be conducted.

The results obtained in this study suggest that although foliar application of wastewater-grown microalgae alone did not significantly enhance yield parameters, its combination with mineral fertilization could potentially improve plant growth and productivity. This synergistic response may arise from the complementary roles of inorganic nutrients and the bioactive compounds present in the microalgae biomass, such as phytohormones, amino acids, and polysaccharides, which can stimulate physiological processes and enhance nutrient uptake efficiency. Supporting evidence from previous studies shows that the integration of microalgae with inorganic fertilizer (NPK) in a 50:50 ratio promoted greater shoot and root development, as well as significant increases in fruit biomass and yield in tomato (*Solanum lycopersicum*) cultivation.^48^ Similar effects were reported for eggplant (*Solanum melongena*), where *Chlorella sp.* combined with organic or inorganic fertilizers achieved yields comparable or superior to conventional fertilization.^49^ These results reinforce that microalgae-based bioinputs can act as complementary agents rather than substitutes for mineral fertilizers, offering a sustainable pathway to optimize nutrient use and crop performance.

Benefits associated with different forms of microalgae application were also reported by Castro et al.^4^ In this study, the authors applied microalgae biofilm to the soil and investigated its effects on the growth of *Pennisetum glaucum*, greenhouse gas emissions, and ammonia volatilization. The results indicated that NH_3_ volatilization losses were lower with the application of biofilm compared to urea. Furthermore, there was an increase in nitrogen levels, organic matter and cation exchange capacity in the soil treated with the biofilm. Although the average dry mass of shoots produced with the microalgae biofilm was 7% lower than that of the control (urea), the results suggest that the microalgae biofilm may offer significant environmental advantages, such as reducing ammonia volatilization and improving soil properties.

de Castro et al.^54^ reported an increase of approximately 10% in the dry weight of corn (*Zea mays* *L*.) plants after the application of a granular fertilizer composed of 12% microalgae biomass and triple superphosphate, compared to the control treatment, which used only triple superphosphate. In addition, an increase in the content and concentration of phosphorus was observed in plants treated with the microalgae-based fertilizer, indicating that the addition of microalgae biomass can enhance the efficiency of phosphorus use in corn cultivation. The pelletization of fertilizer containing mixtures of microalgae biomass and urea in a 50:50 ratio also favors greater corn productivity.^35^

Suchithra et al.^55^ performed a foliar application of *Chlorella vulgaris* in tomato crops (*Solanum lycopersicum* *L*.), using a 100% microalgae biomass extract diluted in water at different doses (25%, 50%, 75%, and 100%). The authors compared foliar application with soil irrigation, using microalgae alone and in combination with cattle manure. The best results in terms of growth, yield, and quality of fruits and seeds were observed with soil irrigation. The parameters analyzed, such as solids, sugars, acids, and proteins, increased with the dose of microalgae. Foliar application, although less effective due to the lower concentration of nutrients, still showed benefits. However, a careful balance is necessary to avoid damage to the leaves. The results indicate the need for further studies to understand the impact of factors such as temperature, light, nutrient concentration, and humidity on the efficiency of foliar applications. It is important to emphasize that new research should be conducted, using different concentrations of the extract and varying the environmental conditions and experimental locations. These investigations could provide more robust information about the potential of the microalgae extract and identify situations in which its application could be more effective.

In addition, foliar application has demonstrated operational and physiological advantages, such as faster nutrient absorption and lower toxicity compared to soil application, as recently evidenced by Tan and Lin^56^ in peach (*Prunus persica* L.) cultivation, where foliar urea sprays effectively induced budbreak while promoting rapid carbohydrate mobilization and maintaining plant safety.

### 3.3 Life Cycle Assessment of Tomato Production

The application of microalgae biomass in tomato production, although only providing part of the nutrient demand, reduced the potential environmental impacts of the process in the 18 midpoint categories. Figure 4 compares the normalized potential environmental impacts for the most relevant categories in tomato production (human carcinogenic toxicity, freshwater ecotoxicity, marine ecotoxicity, water consumption, freshwater eutrophication, and marine eutrophication in descending order), between the baseline scenario and the scenario with microalgae biomass application. Table S2 presents the numerical results of the characterization, comparing the life cycle of tomato production.

**Figure 4:**
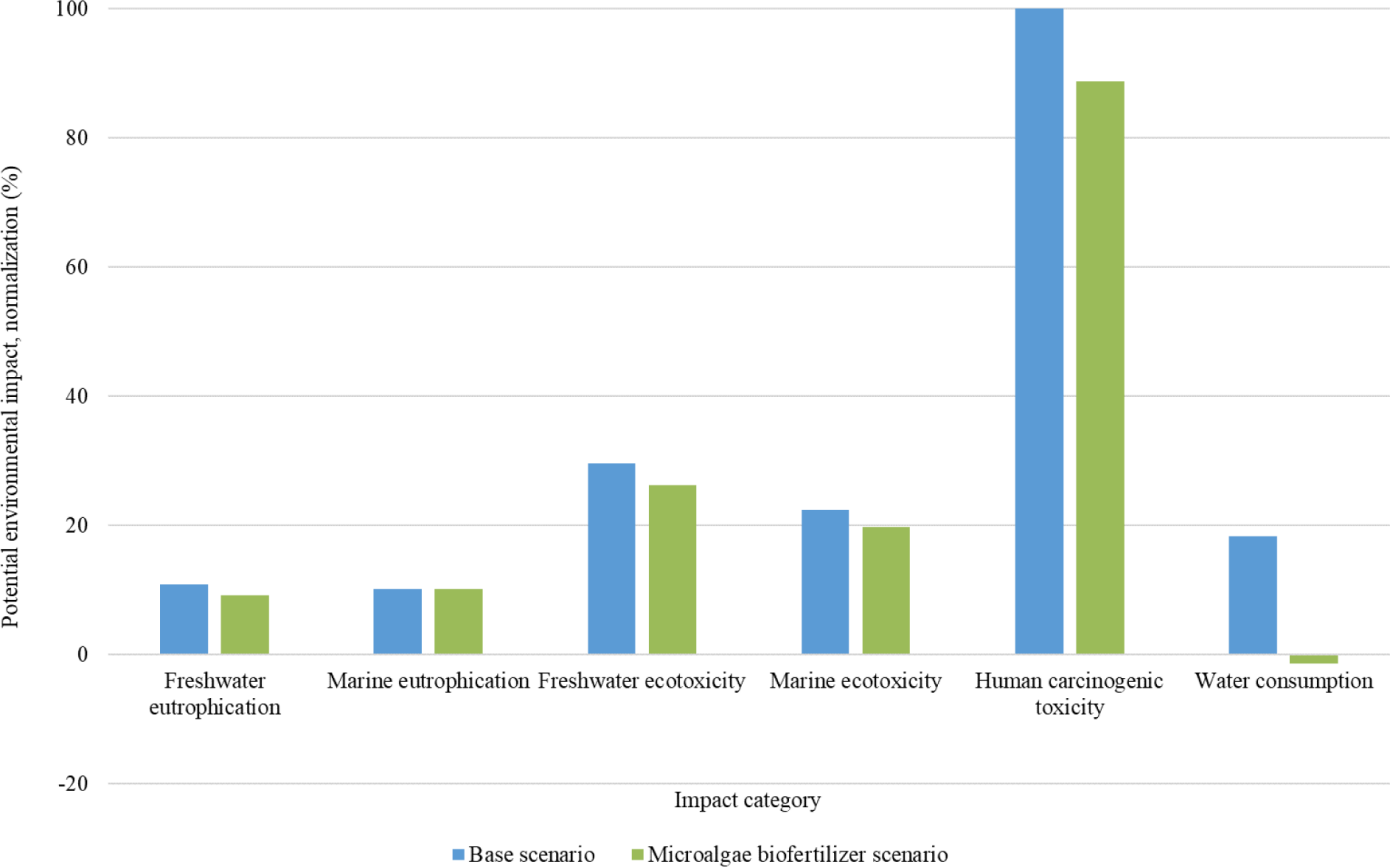
Normalized potential environmental impacts of tomato production in the baseline scenario and in the scenario with microalgae biomass application.

In the scenario using microalgae biomass and treated wastewater, emissions associated with human carcinogenic toxicity, freshwater ecotoxicity, and marine ecotoxicity were reduced by 11% (expressed as kg 1,4-DCB) for each of these categories, based on the production of 1 kg of tomatoes. In freshwater eutrophication, the reduction in emissions was 16%. In addition, this scenario resulted in the avoidance of approximately 2,000 L of freshwater consumption.

These results corroborate those found by Manoukian et al.^17^ who listed the categories of human toxicity and eutrophication as the most common impacts in studies of phosphate fertilizers. This is because the production of mineral phosphate fertilizers has a wide range of environmental impacts that must be considered in LCAs. In addition to the impacts mentioned above, it is known that phosphorus mining has increased the global cycling rate of P deposits to the oceans 4-fold,^57^ with the potential to cause eutrophication. Furthermore, deposits must be mined and processed to transform the ore into bioavailable P fertilizer. The resulting waste may contain potentially toxic trace metals, and the process contributes to greenhouse gas emissions and eutrophication.^17^ In addition, studies that carried out LCAs of similar systems identified the use of nitrogen fertilizers as a hotspot for pollutant emissions.^15,16^ Therefore, it is essential to seek alternatives for the more sustainable production and use of these essential inputs for modern agriculture.

At the end point level (Figure 5), both scenarios still cause damage to the categories of human health, ecosystems, and resources; however, the use of microalgae biomass reduces the total damage by about 25%.

**Figure 5:**
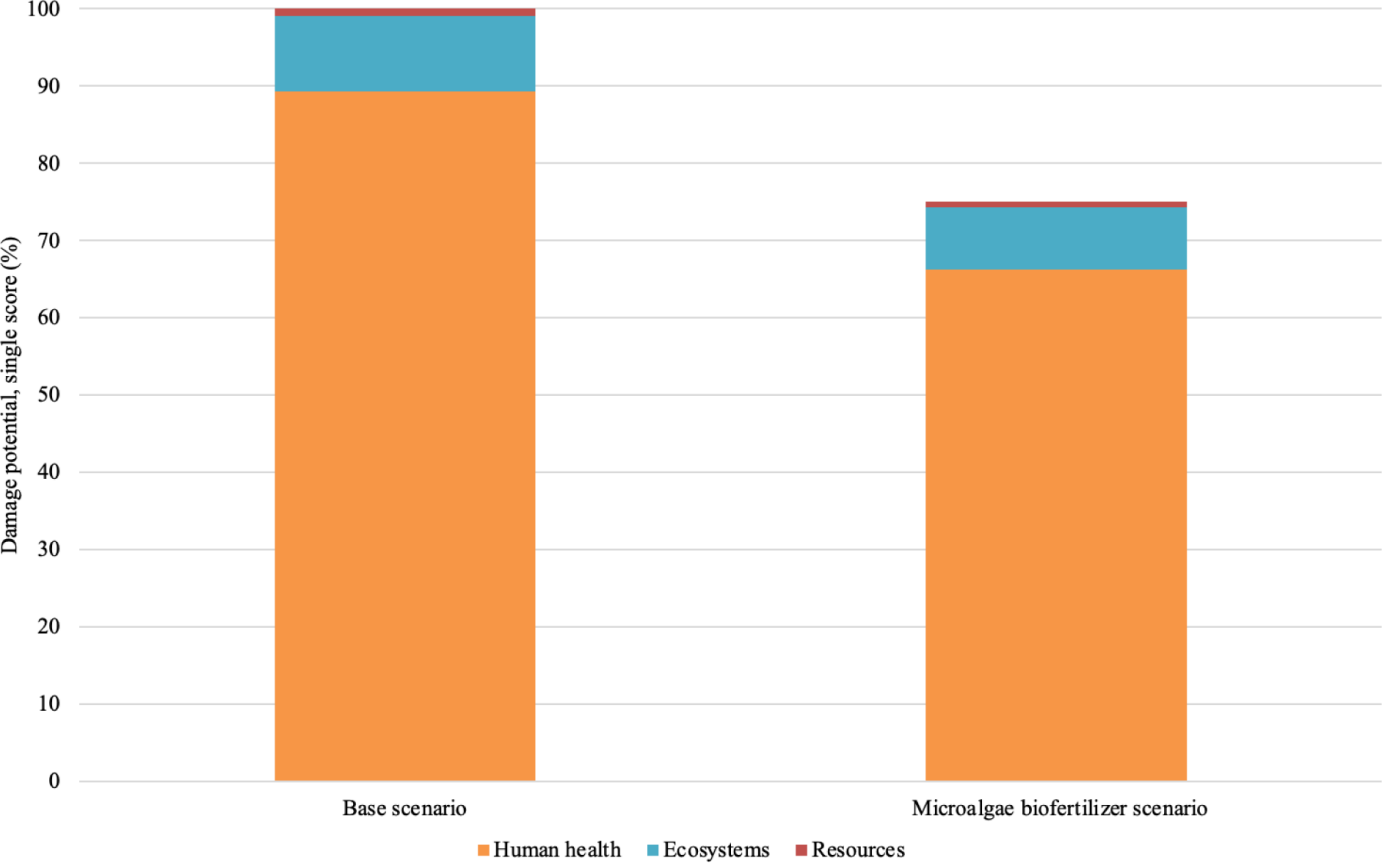
Potential damages from tomato production (end point analysis).

The use of microalgae-based biofertilizers in agriculture, especially in tomato cultivation, has proven to be a promising and environmentally responsible alternative. Although it has not completely met the nutritional needs of this crop, this practice has helped to reduce environmental impacts in the various categories analyzed, especially by reducing dependence on conventional fertilizers, whose production and extraction can be polluting. The improvements observed, such as the reduction in the water footprint and the reduction in emissions associated with toxicity and eutrophication, have highlighted the potential of biofertilizers to mitigate the harmful effects of traditional agricultural systems. However, the challenge of completely replacing the use of mineral fertilizers persists, requiring more research and innovation to increase the effectiveness and reach of microalgae biofertilizers, promoting more sustainable and environmentally beneficial agricultural practices. In addition, it is essential to improve consumers’ social acceptance of the use of products from wastewater treatment.^58^

### 3.4 Challenges and Future Perspectives

The challenges and future prospects of using wastewater-grown microalgae biomass for smart and sustainable agriculture are broad and promising. Field observations from this study indicated a possible reduction in symptoms caused by *Ralstonia solanacearum* in plants treated with microalgae biomass. Although these results were not obtained through a specific phytopathological assay, they suggest a potential role of microalgae in mitigating bacterial wilt. This preliminary evidence, supported by literature describing the antimicrobial activity of microalgae-derived metabolites, underscores the need for targeted experiments under controlled conditions to confirm this effect. Future research should focus on elucidating the mechanisms of action of microalgae against this pathogen, assessing its effectiveness under different growing conditions, dosages, and soil types. Additional trials are also needed to verify whether biomass application has long-term effects on *Ralstonia* control and whether similar outcomes can be achieved in crops other than tomato.

To maximize the applicability of microalgae biomass on a large scale, it is recommended that future studies consider the use of simulation tools, such as Aspen Plus, to simulate and optimize the production process on an industrial scale. Modeling in Aspen Plus would allow assessing technical feasibility and identifying potential bottlenecks in production at larger volumes. After simulating and scaling up the processes, it would be important to carry out an environmental and economic analysis of the increased scale, which would include a detailed assessment of the environmental impacts and costs involved. This step is essential to ensure that the process is economically viable and sustainable, considering aspects such as resource consumption and the potential for reducing environmental impacts, as already observed in the LCA study carried out. It is important to note that LCA results are subject to uncertainty due to variability in inventory data, emission factors, and modeling choices. Although this study does not include a formal uncertainty analysis, future work could incorporate Monte Carlo simulations to quantify confidence intervals and provide a more robust assessment of the results.

Another aspect to be explored in future studies is the use of microalgae biomass as a biofertilizer applied directly to the soil, instead of foliar application. Soil application could offer additional benefits for soil health and promote a more gradual and sustained release of nutrients, contributing to balanced plant growth and potentially improving soil structure. Studies focused on this application could investigate the effects of biomass on increasing soil organic matter, microbiota, and moisture retention, fundamental parameters for sustainable agriculture. By integrating these new research and development approaches, the use of microalgae grown in wastewater as bioinputs could become a viable and beneficial strategy for global agricultural sustainability.

## 4 Conclusion

This study reinforces the potential of microalgae biomass grown in wastewater as a viable and sustainable alternative for plant nutrition in agriculture, particularly in tomato production. The field experiment showed that, although different doses of microalgae biomass did not result in statistically significant postharvest differences, its use as a foliar biofertilizer represents a promising alternative to mineral fertilizers, mainly due to its potential contribution to the control of *Ralstonia solanacearum*. This finding opens new perspectives for future research on the use of microalgae as biological control agents.

The environmental assessment further demonstrated clear advantages of using microalgae biomass as a nutrient and water source, particularly through reduced water use and toxicity impacts. These results highlight the potential of microalgae-based bioinputs to lessen dependence on synthetic fertilizers, mitigate resource depletion, and contribute to more sustainable agricultural systems. Moreover, the improvements evidenced by the LCA may also lead to economic benefits by reducing mineral fertilizer demand and associated production costs, reinforcing the relevance of this approach for both environmental and economic sustainability.

Overall, the study confirms the technical and environmental viability of wastewater-grown microalgae as a nutrient source for sustainable agriculture, offering benefits that extend beyond plant nutrition, including potential biocontrol and environmental impact reduction. To advance these findings, future research should apply process simulation tools (e.g., Aspen Plus) to model industrial-scale production. This approach would allow the inclusion of parameters not addressed here, such as large-scale energy demand, recovery efficiencies, and operational bottlenecks, while enabling a more robust uncertainty analysis. Such simulations are essential to validate the environmental and economic feasibility of scaling microalgae-based fertilizers.

## Supplementary Material



## Notes

Postharvest plant and fruit parameters (Table S1); environmental impact assessment results for tomato production (Table S2) (PDF)
