# Supplementary material for: Microalgae Cultivated in Industrial Wastewater as Agricultural Bioinputs: Technical and Life Cycle Assessment to Support Sustainable Production
Source: ACS Omega. 2025 Nov 24;10(48):59208–18. doi: 10.1021/acsomega.5c08477 (PMC12771248; doi:10.1021/acsomega.5c08477)
Supplement: Supplementary file 1 [file ao5c08477_si_001.pdf]

Microalgae cultivated in industrial wastewater as agricultural bioinputs: technical and life cycle assessment to support sustainable production

Karoline Matiello Almeida[a], Bianca Barros Marangon[b], Vinícius José Ribeiro[b], Jackeline de Siqueira Castro[b], Juscimar da Silva[c], Edson Marcio Mattiello[a], Andreia Aparecida de Sousa Silva[c], Eduarda Cristina Moreira Silva[c], Maria Lúcia Calijuri[b]*

[a] Department of Soils and Plant Nutrition, Federal University of Viçosa (Universidade Federal de Viçosa), Viçosa, Minas Gerais 36570-900, Brazil.

[b] Department of Civil Engineering, Center for Advanced Research in Microalgae, Federal University of Viçosa (Universidade Federal de Viçosa), Viçosa, Minas Gerais 36570-900, Brazil.

[c] Brazilian Agricultural Research Corporation – EMBRAPA Vegetables, Brasília, Distrito Federal 70359-970, Brazil.

*Corresponding author: jackeline.castro@ufv.br; +5534999113838

Keywords: microalgae biomass; resources recovery; foliar fertilizer; carbon footprint; circular economy.

Table S1. Post-harvest parameters of plants and fruits analyzed.

Treat.	SPAD	Fruit length (mm)	Fruit diameter (mm)	% citric acid	° BRIX	pH	Weight (kg)	Number of fruits
T1	46.88 ± 3.4 a	74.75 ± 3.5 a	42.46 ± 2.1 a	1.04 ± 0.18 a	4.20 ± 0.52 a	4.57 ± 0.09 b	7.28 ± 0.96 a	103 ± 17.9 ab
T2	51.92 ± 3.1 a	73.65 ± 7.0 a	44.10 ± 1.5 a	0.87 ± 0.15 a	3.90 ± 0.32 a	4.69 ± 0.07 a	6.81 ± 0.71 ab	116 ± 8.67 a
T3	48.45 ± 4.3 a	75.17 ± 3.7 a	43.57 ± 0.9 a	0.83 ± 0.13 a	4.25 ± 0.71 a	4.80 ± 0.03 a	6.37 ± 0.79 ab	101 ± 10.4 ab
T4	51.79 ± 2.9 a	79.41 ± 2.2 a	44.20 ± 2.1 a	1.02 ± 0.18 a	4.33 ± 0.08 a	4.68 ± 0.04 ab	6.96 ± 1.23 ab	108 ± 18.5 ab
T5	51.27 ± 2.4 a	73.53 ± 6.9 a	43.36 ± 4.1 a	0.82 ± 0.17 a	4.38 ± 0.71 a	4.72 ± 0.02 a	6.05 ± 1.54 b	90 ± 8.6 b

Note: Equal letters indicate that there was no statistical difference between the means at the 10% level of the Tukey test. Bars indicate standard deviation.

Table S2. Potential environmental impact of tomato production.

Impact category	Unit	Base scenario	Microalgae biofetilizer scenario
Global warming	kg CO_2 eq	9.59E-02	8.57E-02
Stratospheric ozone depletion	kg CFC11 eq	5.54E-07	5.49E-07
Ionizing radiation	kBq Co-60 eq	2.27E-03	1.06E-03
Ozone formation, Human health	kg NOx eq	4.19E-04	3.99E-04
Fine particulate matter formation	kg PM2.5 eq	1.88E-04	1.69E-04
Ozone formation, Terrestrial ecosystems	kg NOx eq	4.25E-04	4.05E-04
Terrestrial acidification	kg SO_2 eq	6.27E-04	5.90E-04
Freshwater eutrophication	kg P eq	3.45E-05	2.90E-05
Marine eutrophication	kg N eq	2.28E-04	2.28E-04
Terrestrial ecotoxicity	kg 1,4-DCB	3.44E-01	3.13E-01
Freshwater ecotoxicity	kg 1,4-DCB	3.62E-03	3.21E-03
Marine ecotoxicity	kg 1,4-DCB	4.72E-03	4.18E-03
Human carcinogenic toxicity	kg 1,4-DCB	5.00E-03	4.45E-03
Human non-carcinogenic toxicity	kg 1,4-DCB	1.83E-01	1.73E-01
Land use	m^2a crop eq	1.50E-01	1.50E-01
Mineral resource scarcity	kg Cu eq	6.21E-04	5.61E-04
Fossil resource scarcity	kg oil eq	2.46E-02	2.12E-02
Water consumption	m^3	2.38E-02	-1.77E-03
